# The Identification of Culicine Mosquitoes in the Shadegan Wetland in Southwestern Iran

**DOI:** 10.1673/031.012.10501

**Published:** 2012-09-04

**Authors:** S. Navidpour, B. Vazirianzadeh, R. Harbach, E. Jahanifard, S. A. Moravvej

**Affiliations:** ^1^ Department of Medical Entomology, Centre of Arthropoda Research, Razi Vaccine and Serum Research Institute, Ahvaz, Iran; ^2^ Department of Medical Entomology and Vector Control, School of Public Health, and Infectious and Tropical Diseases Research Centre, Ahvaz Jundi-Shapour University of Medical Sciences, Ahvaz, Iran; ^3^ Department of Entomology, Natural History Museum, London, UK; ^4^ Department of Medical Entomology and Vector Control, School of Public Health, Ahvaz Jundi-Shapour University of Medical Sciences, Ahvaz, Iran

**Keywords:** Culicinae, fauna, Khouzestan Province

## Abstract

In order to study the culicine mosquito fauna (Diptera: Culicidae: Culicinae) of the Shadegan wetland in southern Khouzestan Province of Iran, sampling was carried out using hand catch, total catch, and New Jersy light traps, from October 2008 to March 2009. A total of 2664 culicine mosquitoes were captured. Three genera and five species were identified, including *Culex pipiens* L., *Cx. tritaeniorhynchus* Giles, *Cx. sinaiticus* Kirkpatrik, *Cx. modestus* Ficalbi, *Ochlerotatus caspius* Pallas and a *Culiseta* species. All of these species, reported for the first time, were from the Shadegan wetland and Khouzestan Province, and some are medically important.

## Introduction

The *Culex* fauna of southwestern Asia was poorly known before Harbach (1988) published his study on the 20 species of subgenus *Culex* that live in southwestern Asia and Egypt. However, many faunistic studies of medically important species in Iran have been conducted by many investigators. For example, Mattingly and Knight (1956), Senevet and Andarelli (1959), Gutsevitch et al. (1974), and Harbach (1988) worked on the mosquitoes of certain countries and specific parts of the region. In 1986, Zaim and Cranston (1986) published a checklist and keys to the Culicinae of Iran, including 31 species in four genera. More faunistic data are available for the genus *Culex* than for other culicine mosquitoes in Iran, which were described by Lotfi (1970, 1973, 1976) along with keys and information about the biology of species of *Culex*. The most recent studies on Iranian mosquitoes were conducted by Azari-Hamidian (2007).

Azari-Hamidian et al. (2002) recorded four species of tribe Aedini from Guilan Province in the north of Iran: *Ochlerotatus caspius, Oc. echinus, Oc. geniculatus*, and *Aedimorphus vexans*. Azari-Hamidian et al. (2003) identified three species of the genus *Culiseta* from northern Iran: *Cs. longiareolata, Cs. morsitans*, and *Cs. annulata*, with *Cs. morsitans* of subgenus *Culicella* reported for the first time in Iran. In another study, Azari-Hamidian et al. (2005) recorded nine *Culex* species (*Cx. bitaeniorhynchus, Cx. deserticola, Cx. laticinctus, Cx. perexiguus, Cx. pipiens, Cx. quinquefasciatus, Cx. sinaiticus, Cx. theileri* and *Cx. tritaeniorhynchus*), and two other culicine species (*Cs. longiareolata* and *Uranotaenia unguiculata*) from Kerman, central east Province of Iran. According to the latest research by Azari-Hamidian (2007), the mosquito fauna of Iran includes 64, species and three subspecies belonging to seven genera.

Because of the medical importance of malaria, the majority of mosquito studies in Iran during the past four decades have focused on anopheline mosquitoes. The majority of works on anophelines in Iran are referenced by Shahgudian (1960).

Some arboviral and parasitic diseases are transmitted by culicine mosquitoes in Iran, including West Nile and Sindbis viruses, *Dirofilaria immitis* (dog heart worm), and *Dirofilaria repens* (Sadigian 1969; Naficy and Saidi 1970; Siavashi and Massoud 1995; Maraghi et al. 2006). Outbreaks of culicine-borne arboviral diseases in the World Health Organization (2004) Eastern Mediterranean region, which includes Iran, are possible, and this provides the motivation for more study on culicine mosquitoes. The studies of Zaim and Cranston (1986) were conducted during a time of war between Iran and Iraq. Since then, the mosquito fauna of much of the southwest of Iran, including the Shadegan wetland, has received little attention. Because of this lack of attention, the major aim of this study was to identify the culicine fauna in the region of the Shadegan wetland.

## Materials and Methods

Adult mosquitoes were collected in the Shadegan wetland (48° 66′ E, 30°, 64′ N) from October 2007 to October 2008 using aspirators, which was the most effective method for indoor collections of certain domestic mosquitoes. Insect nets were used to collect the mosquitoes outdoors, and New Jersey light traps were used in rural environments where there were few competing light sources. The sampling was conducted from sunset to 22:00 for all mentioned methods.

**Table 1.**
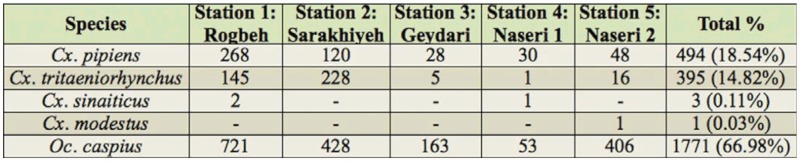
Frequencies of the adult mosquitoes collected in the Shadegan wetland October 2007 to March 2008.

Specimens were collected from five different rural parts of the Shadegan wetland: Sarakhiyeh (48° 45′ E, 30° 32′ N), Naseri 1 (48° 37′ E, 30° 38′ N), Naseri 2 (48° 37′ E, 30° 38′ N), Rogbeh (48° 33′ E, 30° 32′ N), and Geydari (48° 40′ E, 30° 40′ N). Mosquitoes were pinned, and identified to genus and species using the keys of Shahgudian (1960), Zaim and Cranston (1986), Harbach (1988) and Darsie and Samanidou-Voyadjoglou (1997). Abbreviations of mosquito genera are based on Reinert (2009).

All specimens were deposited in the museum of the Department of Medical Entomology, Centre of Arthropoda Research, Razi Vaccine and the Serum Research Institute, Department of Medical Entomology and Vector control, School of Public Health, Centre of Infectious and Tropical Diseases Research Centre, Ahvaz Jundishapour University Medical Sciences (AJUMS), Ahvaz, Iran.

## Results

A total of 2664 culicine mosquitoes representing six species of two genera were collected during the study period, including *Culex pipiens* L., *Cx. tritaeniorhynchus* Giles, *Cx. sinaiticus* Kirkpatrik, *Cx. modestus* Ficalbi, and *Ochlerotatus caspius* Pallas (Table 1). A single unidentifiable specimen of *Culiseta* was collected, but it is not listed in Table 1.

## Discussion

The Shadegan wetland covers an area of 296,000 hectares. The area includes many suitable habitats for culicine larvae, but no previous information was available for culicine mosquitoes in this region of southwestern Iran. Consequently, the species reported in this study are new records for the Shadegan wetland.

*Cx. pipiens* was reported as a complex species in the studies of mosquitoes of the Middle East and Iran by Zaim and Cranston (1986), Harbach (1988), and Azari-Hamidian (2007), and also in Iraq by Ibrahim et al. (1983). Based on provenance and morphological identification using the keys noted above, only *Cx. pipiens* is present in the Shadegan wetlands. There is no evidence for the presence of *Cx. quinquefasciatus* Say, which is known to occur in Iraq.

*Cx. pipiens* is reported to comprise 27.1% of mosquitoes encountered in Kerman Province of eastern Iran (Azari-Hamidian et al. 2005). This species comprised 18.5% of the culicine mosquitoes collected in the Shadegan region, the second most common mosquito after *Oc. caspius*. This agrees with the study of Azari-Hamidian et al. (2003) in the Kerman Province.

*Cx. tritaeniorhynchus* is the second most abundant species of *Culex*, and the third most abundant culicine species, comprising 14.8% of the specimens collected during the study. This species is known to occur in other areas of Iran (Zaim and Cranston 1986; Harbach 1988; Azari-Hamidian 2007) and Iraq (Ibrahim et al. 1983; Harbach 1988). *Cx. pipiens* is a cosmopolitan species whereas *Cx. tritaeniorhynchus* is restricted to the Palaearctic (southern Asia), Afrotropical, and Oriental regions (Zaim and Cranston 1986). These two species are much more abundant in the Shadegan region than *Cx. sinaiticus* (0.11%) and *Cx. modestus* (0.03%), and are also more common than *Cx. theileri* in the Hovizeh region (Vazirianzadeh and Navidpour 2008) in southwestern Iran. This also agrees with Azari-Hamidian et al. (2005), who reported a similar relative abundance of culicine mosquitoes in the Kerman area: *Cx. tritaeniorhynchus* (10.8%), *Cx. sinaiticus* (6.3%) and *Cx. theileri* (3.8%). Whereas *Cx. sinaiticus* and *Cx. modestus* have the lowest populations in of the Shadegan region, *Cx. laticinctus* (0.3%) is the least abundant species in Kerman Province (Azari-Hamidian et al. 2005).

*Oc. caspius* is the most abundant species collected in the Shadegan wetland, compromising 66.98% of the culicine population. This agrees with the study of Zaim et al. (1984), who showed that this species is one of the most abundant species of Culicidae because its larval stage is adapted to thrive in saline marshlands. The Shadegan wetland is becoming more saline as a result of drainage from sugar cane farms in recent years (Vazirianzadeh and Navidpour 2008). *Oc. caspius* has been collected in many provinces of Iran (Zaim et al. 1984), but not in the southern province of Kerman (Azari-Hamidian et al. 2005), which shows that the ecology of the habitat plays a principal role in the occurrence and abundance of mosquitoes. *Oc*. is widely distributed in coastal and saline areas of the Palaearctic Region.

Differences between the results of this study and other studies are probably due to the use of different sampling methods, but may also be due to sampling in different years and different times of the year. The results reported here are based only on adult sampling.

The species collected in the Shadegan wetland are all of potential medical importance. *Ochlerotatus* are capable of transmitting West Nile fever and other arboviruses. This is very important in the Shadegan area, where *Oc. caspius* is the most abundant population of culicine mosquitoes. It is an anthropophilic mosquito that bites during the day and is capable of flying long distances (Zaim et al. 1984). The presence of *Cx. pipiens* and *Cx. tritaeniorhynchus* in the Shadegan region is significant because both of these species are vectors of various encephalitis viruses, including West Nile fever (both species) and Japanese encephalitis viruses (*Cx. tritaeniorhynchus*) (Zaim et al. 1984; Azari-Hamidian et al. 2005; Azari-Hamidian 2007). *Cx. pipiens* is also a vector of the parasitic worm that causes dirofilariasis in Khouzestan Province of southwestern Iran, where the Shadegan wetland is located (Maraghi et al. 2006).
